# Relationship between depressive symptom severity and emergency department use among low-income, depressed homebound older adults aged 50 years and older

**DOI:** 10.1186/1471-244X-12-233

**Published:** 2012-12-26

**Authors:** Namkee G Choi, C Nathan Marti, Martha L Bruce, Mark E Kunik

**Affiliations:** 1 The University of Texas at Austin, Austin, TX, USA; 2 Weill Cornell Medical College, White Plains, NY, USA; 3 VA HSR&D Houston Center of Excellence, Michael E. DeBakey VA Medical Center, Houston, TX, USA; 4 Baylor College of Medicine, Houston, TX, USA; 5 VA South Central Mental Illness Research, Education and Clinical Center, Houston, TX, USA

**Keywords:** Homebound older adults, Depression, Emergency department

## Abstract

**Background:**

Previous research found a high prevalence of depression, along with chronic illnesses and disabilities, among older ED patients. This study examined the relationship between depressive symptom severity and the number of ED visits among low-income homebound older adults who participated in a randomized controlled trial of telehealth problem-solving therapy (PST).

**Methods:**

The number of and reasons for ED visits were collected from the study participants (n=121 at baseline) at all assessment points—baseline and 12- and 24-week follow-ups. Depressive symptoms were measured with the 24-item Hamilton Rating Scale for Depression (HAMD). All multivariable analyses examining the relationships between ED visits and depressive symptoms were conducted using zero-inflated Poisson regression models.

**Results:**

Of the participants, 67.7% used the ED at least once and 61% of the visitors made at least one return visit during the approximately 12-month period. Body pain (not from fall injury and not including chest pain) was the most common reason. The ED visit frequency at baseline and at follow-up was significantly positively associated with the HAMD scores at the assessment points. The ED visit frequency at follow-up, controlling for the ED visits at baseline, was also significantly associated with the HAMD score change since baseline.

**Conclusions:**

The ED visit rate was much higher than those reported in other studies. Better education on self-management of chronic conditions, depression screening by primary care physicians and ED, and depression treatment that includes symptom management and problem-solving skills may be important to reduce ED visits among medically ill, low-income homebound adults.

**Trial registration:**

ClinicalTrials.gov Identifier: NCT00903019

## Background

Older adults in North America use emergency departments (EDs) at a higher rate than younger adults [1-6]. Previous studies also found that a significant proportion of older adults released from an ED make return visits and return frequently [7,8]. Common diagnoses among older ED patients included injuries caused by falls; acute cerebrovascular accidents; infections (including pneumonia, bronchitis, and urinary tract infections), abdominal disorders, and dehydration [1,3]. Chronic illnesses—especially cardiac, respiratory, and diabetic diseases—and high levels of comorbidities, including cognitive impairment and depression, and disabilities are significant predictors of ED visits among older adults [1-4,7-13]. In addition to these clinical characteristics, being Black, having a low income and Medicaid coverage, being uninsured, and/or not having a primary care physician (PCP) were associated with a higher level of ED visits [4,14]. However, compared to younger ones, older ED patients more often had a PCP and were referred to the ED by their PCP [1].

Compared to their nondepressed peers, depressed older adults tend to use both ambulatory and inpatient healthcare services at a significantly higher rate even after adjusting for chronic medical illness, and their high rates of ED visits and return visits have been well documented [7,9,11,15-18]. Depression in older adults especially with chronic medical conditions can result in increased numbers of ED visits because depression (1) tends to amplify both symptoms of the physical illness and physical symptoms, including pain and discomfort, associated with other body organ systems; (2) has been shown to adversely impact self-management/self-care of chronic illness by its adverse effect on memory, energy, sense of self-efficacy, and adherence to medication, diet, and exercise regimens; and (3) adversely affects satisfaction with care and may add a degree of urgency to the pursuit of help [19,20]. Increased severity of medical illness could also lead to increased symptoms of depression, and untreated depression or worsening depressive symptoms may mimic or exacerbate the somatic symptoms associated with other chronic medical conditions [9]. Conversely, treated depression or reduced depressive symptoms may contribute to positive health perceptions and a decrease in ED visits.

Despite the high volume of extant research on depressed older adults’ ED visits, few studies examined the longitudinal relationship between depressive symptoms and the frequency of ED visits. The primary aim of the present study was to examine (1) whether depressive symptom severity was associated with the frequency of ED visits at two different points of time among low-income, depressed homebound older adults aged 50 years and older; and (2) whether changes in depressive symptom severity overtime was associated with the changes in the frequency of ED visits and return visits. The older adults’ self-reported reasons for their ED visits were also presented. The study subjects were residents of central Texas in the United States who participated in a randomized controlled trial (RCT) of a short-term psychotherapy—telehealth-delivered or in-person problem-solving therapy (PST) compared to telephone support call—for low-income homebound older adults with moderately severe or severe depressive symptoms at baseline. The longitudinal data on the changes, or lack thereof, in the study subjects’ depressive symptoms and ED visits during the study period offered a great opportunity to examine longitudinal relationship between depressive symptoms and the frequency of ED visits among these older adults who were largely underrepresented in previous research on older adults’ ED use.

According to the 2011 United States census data, of 40 million non-institutionalized adults age 65 years and older, 23.5% (9.2 million) had ambulatory disability/activity limitation and about 10% were considered homebound [21-23]. The rates of major depression and clinically significant depressive symptoms have been found to be twice as high in homebound older adults as in their age peers without mobility impairment [24-27]. Homebound older adults, often suffering from multiple chronic illnesses and depression, are frequent users of intensive and costly healthcare services including rapidly increasing home healthcare services in the United States [23,28]. Low-income, depressed homebound older adults are especially likely to be frequent users of EDs, given their precarious physical and functional health conditions comorbid with depression and other multiple life stressors associated with lack of financial resources. These older adults tend to be socially isolated and have difficulty managing their physical and mental health conditions due to many other competing life demands (e.g., housing issues, financial worries, family relationship issues) and, oftentimes, lack of information and knowledge needed for treatment adherence and self-management of their chronic diseases [29,30].

Employing Andersen’s behavioral model of health services use [31] as the conceptual framework, the study hypotheses were as follows: (H1) higher depressive symptoms at baseline would be associated with a higher frequency of ED visits during the six months prior to baseline; (H2) higher depressive symptoms at 24-week follow-up would be associated with a higher frequency of ED visits during the 24 weeks after baseline; and (H3) reduced depressive symptoms 24 weeks after baseline would be significantly associated with a lower frequency of ED visits during that period, controlling for baseline number of ED visits and predisposing, enabling, and other need factors.

## Methods

### Setting and participants

Following the definition of “homebound” older adults in Medicare [32], the term *homebound adults* in this study referred to older individuals who, due to medical conditions and/or mobility-affecting impairments, cannot freely leave their home and who require help in doing so. Case managers at a large Meals on Wheels program and other social service agencies serving low-income, homebound adults age 50 years and older in central Texas referred to the project those who scored 10 or higher on the PHQ-9 [33,34] or showed other signs of depression. We included the 50–64 age group since our previous study [35] found that a larger proportion of homebound individuals in this age group than those in the 65+ age group suffered from depression and were frequent users of healthcare services. Referred individuals who provided informed consent were administered the 24-item Hamilton Rating Scale for Depression (HAMD) and the *DSM-IV-TR* diagnostic interviews for depression. For consistency with other RCTs of depressed older adults in primary care or home healthcare settings in the United States [36-38], those whose HAMD scores were 15 or higher were included in the RCT. The mean 24-item HAMD score for home healthcare patients with major depression at baseline was about 15 [36]. In the multisite, randomized trial known as PROSPECT (Prevention of Suicide in Primary Care Elderly: Collaborative Trial), the lower bound of the 90% confidence interval of the 24-item HAMD for patients with major depression was also about 15 [37,38].

The exclusion criteria were (1) high suicide risk; (2) dementia (assessed with the Mini-Cog [39]); (3) bipolar disorder; (4) 12-month or lifetime psychotic symptoms or disorder; (5) presence of co-occurring alcohol and/or other addictive substance abuse; and (6) current involvement in psychotherapy. Those who had been on antidepressants more than two months but who showed significant depressive symptoms were not excluded. Written informed consent, approved by the first author’s institutional review board, was obtained from each participant after the study procedures had been fully explained and before any screening and baseline assessments were conducted.

Of 186 referrals received during the 24-month recruitment and enrollment period, 124 met the inclusion criteria, and 121 who agreed to participate in the study were randomly assigned to three groups—telehealth-delivered PST; in-person PST and telephone support calls (attention control) with each intervention consisting of six weekly sessions. Of the 121 who began participation in the study, 14 (11.6%) dropped out during the intervention phase and 11 (9.1%) dropped out during the follow-up period. Fifteen participants did not complete their 12-week follow-up assessment and 25 did not complete their 24-week follow-up assessment. Attrition was due mostly to deteriorating health that resulted in hospitalization, nursing home placement, and death; however, the baseline demographic, family support, and clinical characteristics (including depressive symptom severity and number of chronic medical illnesses); intervention group assignment; and self-reported frequency of ED visits of the dropouts (*n* = 25) did not differ significantly from the characteristics of those who completed the 24-week follow-up (*n* = 96).

### Ethics

The protocol for this research was approved by the Institutional Review Board of the University of Texas at Austin, and the research was carried out in compliance with the United States National Institute of Health regulations, policy, and guidelines for human subject research (http://grants.nih.gov/grants/policy/hs/hs_policies.htm).

### Measures

#### ED visits

The number of and reasons for ED visits were collected from the participants at all assessment points—baseline and 12- and 24-week follow-ups. At baseline, they were asked to report ED visits during the preceding six months. At 12-week follow-up, they reported the ED visits since baseline, and at 24-week follow-up, they reported the ED visits since the 12-week follow-up. The sum of the ED visits reported at these two follow-up assessments was the frequency of ED visits between baseline and 24-week follow-up. One study [40] of self-reported health services use among urban older adults (≥60 years) found that 28.1% of those who had visited an ED in the preceding 12 months failed to report the episode recorded in an electronic medical record system, but the study could not identify any patient characteristics associated with the underreport. In the present study, we asked the participants about their ED visits in the preceding six (at baseline) or three (at follow-ups) months, allowing them relatively short periods of recall. At baseline, a consistent pattern we found was that those who had not visited the ED or who had visited it only once were highly confident of their recall accuracy, while some of those who had visited the ED multiple times had difficulty recalling the exact numbers (e.g., “I am not sure if it was three or four times”). With respect to the ED visit data at follow-ups, we cross-referenced them with the PST or telephone support call progress notes (weekly sessions plus monthly booster calls) in which any ED visit was recorded. Only one discrepancy from cross-referencing was found, and it was handled by calling the participant for clarification.

#### Predisposing factors

These were gender, age groups (50–59, 60–69, and 70+), and race/ethnicity (African American, Hispanic, and non-Hispanic White).

#### Enabling factors

These were Medicaid coverage (yes = 1, no = 0) and family support, which was measured by the 6-item family support scale of the Lubben Social Network Scale Expanded [41] (LSNS-E). This scale has been frequently used to measure the size of older adults’ social support networks and their perceived and actual levels of social support from these networks. Cronbach’s α for the study participants was .73 at baseline.

#### Depressive symptoms as a need factor

Depressive symptoms were measured with the 24-item HAMD. It consists of the GRID-HAMD-21 structured interview guide [42] augmented with three additional items assessing feelings of hopelessness, helplessness, and worthlessness, with specific probes and follow-up questions developed by Moberg et al. [43]. We treated depressive symptoms as a need factor, because depression, as discussed, tends to amplify both symptoms of the physical illness and physical symptoms, adversely impact self-management of chronic illness, and adversely affect satisfaction with care and may add a degree of urgency to the pursuit of help.

#### Other need factors

The number of diagnosed chronic medical conditions was reported by the participants with these questions at baseline: “Have you ever been diagnosed by a healthcare professional as having any of the following conditions?” and “Is [the condition] still a problem?” Included conditions were arthritis, hypertension, diabetes, heart disease, stroke, lung disease, cancer, and kidney disease. Only conditions that were still problems were counted. Pain frequency at baseline was measured with two questions: (1) Have you had chronic pain in any part of your body during the past six months? and (2) How would you rate the frequency of your pain on a 10-point scale, with 1 being very infrequently (like once a week or less often) and 10 being all the time? Those who reported having no pain were assigned a score of 0.

### Analysis

All analyses were performed using SAS v. 9.2 (SAS Corp., Cary, NC). Prior to assessing bivariate associations between the frequency of ED visits and participants’ predisposing, enabling, and need characteristics, the probability distributions of the outcomes were assessed. Due to non-normal distributions, robust regression, which is robust to heteroscedastic error and outliers, was used for all group comparisons using SAS PROC ROBUSTREG. H1 and H2 were tested using zero-inflated Poisson regression models using SAS PROC GENMOD, with the frequency of ED visits at baseline and at 24-week follow-up, respectively, as the dependent variable, and with predisposing, enabling, and need factors (depression severity at baseline or 24-week follow-up and the number of medical conditions and pain frequency at baseline) evaluated as covariates. H3 was also tested using zero-inflated Poisson regression analysis, with the ED visits at 24-week follow-up as the dependent variable and the difference between the 24-week follow-up HAMD score and the baseline HAMD score as the predictor variable, controlling for the baseline HAMD score and other covariates. Prior to implementing the models, we assessed possible overdispersion of the count outcome data by testing whether the negative binomial dispersion parameter was significantly different from zero. Because it was not, we used a Poisson, rather than a negative binomial, distribution to model the count outcome variables.

Because there were a large number of variables relative to the sample size and all the variables could potentially be included in both the logistic and count portions of the model, a variable reduction strategy was employed. Guidelines provided by Hosmer and Lemeshow [44] were used to minimize the number of variables included in the logistic portion of the model, which is particularly sensitive to overparameterization. In order to avoid inflated standard errors and numerically unstable results, Hosmer and Lemeshow recommend having at least 10 cases per event, which limited us to seven variables (69 participants had ED visits during the pretest period) and two variables (23 participants had ED visits during the pretest period) for the posttest period. Each of these potentially confounding variables was evaluated using the following steps: (a) bivariate relationships between the covariates and the outcomes were examined, (b) variables with p values below α = .25 in the first step were retained, and (c) retained variables were included in a multivariable model and a minimally adequate set of covariates was established using a likelihood ratio test and the Akaike information criterion (AIC), using guidelines from Burnham and Anderson [45]. Variables below the α = .25 in the count portion of the model and the full and reduced model were compared using a likelihood ratio test and the AIC to assure that the more parsimonious model was indeed a better fit to the data.

## Results

### Participant characteristics

Table 1 shows that the participants were diverse in age, that 59.7% were African American or Hispanic, and that 84.4% had annual family income ≤$25,000. On average, they had three chronic medical conditions. All had a PCP or regular source of care (with 91.7% enrolled in Medicare and/or Medicaid and another 5% with Department of Veterans Affairs insurance or employer-originated private insurance). Further analysis showed no difference in baseline depressive symptom severity by any demographic characteristics, level of family support, number of chronic conditions, pain frequency, or intervention group assignment (tele-PST, in-person PST, and telephone support call).

**Table 1:** Demographic and clinical characteristics of participants at baseline (N = 121)

Age, mean (SD)	65.21 (+9.22)
(range: 50-89)	
Age group (n, %)	
50-59	38 (31.4)
60-69	48 (39.7)
70+	35 (28.9)
Gender (n, %)	
Male	27 (22.3)
Female	94 (77.7)
Race/ethnicity (n, %)	
Non-Hispanic White	50 (41.3)
Black	41 (33.9)
Hispanic	30 (24.8)
SCID diagnosis (n, %)	
Major Depressive disorder	81 (67.0)
Depressive disorder, NOS	35 (28.9)
Dystymia	5 (4.1)
Living arrangement (n, %)	
Living alone	77 (63.6)
Not living alone	44 (36.4)
Family income (n, %)	
<= 15,000	77 (63.6)
15,001-25,000	25 (20.7)
25,001-50,000	12 (9.9)
Don’t know/refused	7 (5.8)
Insurance coverage (n, %)	
Medicare	96 (79.3)
Medicaid	40 (33.1)
Private insurance (past employer-provided)	18 (14.9)
Veterans administration insurance	14 (11.6)
Family support^1^	16.05 (+5.97)
(range: 0-26)	
Depression severity (HAMD score), mean (SD)	24.55 (+6.62)
(range: 15-42)	
No. of diagnosed medical conditions, mean (SD)	3.19 (+1.54)
Chronic pain (n, %)	
Yes	106 (87.5)
No	15 (12.4)
Pain frequency^2^	7.75 (3.40)
RCT group (n, %)	
Tele-PST	43 (35.5)
In-person PST	42 (34.7)
Telephone support call	36 (29.8)

### ED visits, depressive symptoms, and reasons

Table 2 shows that at baseline, 43% of the participants reported no ED visits during the preceding six months, 22.3% reported 1 visit, 12.4% reported 2 visits, and 21.4% reported 3–9 visits. Of 68 participants who had had any ED visit, 39.1% (*n* = 37) reported 1 visit, 21.7% (*n* = 15) reported 2 visits, and 39.1% reported 3–9 visits; thus, almost 61% made return visits. At 24-week follow-up, 49.6% reported no ED visit since baseline, 16.5% reported 1 visit, 5.8% reported 2 visits, and 7.5% reported 3–8 visits. Of the 36 participants who had any ER visit, 55.6% (*n* = 20) had only 1 visit, 19.4% (*n* = 7) had 2 visits, and 25% (*n* = 9) had 3–8 visits. The total number of ED visits reported was 170 (for an average of 2.5 visits per visitor) at baseline and 88 (for an average of 2.4 visits per visitor) at 24-week follow-up. The baseline HAMD scores were not normally distributed according to the Shapiro-Wilk test (W = 0.94, p < .001); thus, comparisons between ED visits and HAMD scores were assessed using robust regression. The HAMD scores for those who had had 3+ ED visits at baseline were significantly higher than the scores of those who had had no visits (χ^2^ = 14.73, p < .001), those who had had one visit (χ^2^ = 5.07, p = .024), and those who had two visits (χ^2^ = 7.82, p = .005). On the other hand, the HAMD scores for those who had had 3+ ED visits at 24-week follow-up were not different from the HAMD scores for those who had had no visits (χ^2^ = 1.36, *p* = .243), those who had had one visit (χ^2^ = 1.39, p = .238), and those that had two visits (χ^2^ = 1.09, p = .300).

**Table 2:** ED visit frequency reported at baseline and follow-up and HAMD score

**6 months prior to baseline (n=121)**			**24-weeks after baseline (n=96)**		
**ED frequency**	**n (%)**	**HAMD scores**	**ED frequency**	**n (%)**	**HAMD score**
0	52 (43.0)	23.04+6.51	0	60 (49.6)	14.08+7.86
1	27 (22.3)	24.70+6.42	1	20 (16.5)	14.45+8.60
2	15 (12.4)	22.60+5.34	2	7 (5.8)	21.71+9.55
3+	26 (21.4)^1^	28.37+6.39	3+	9 (7.5)^2^	17.33+4.44

^1^The range was 3 to 9; ^2^The range was 3 to 8.

Table 3 shows that 33.3% of participants who provided 24-week follow-up data had no ED visits at both baseline and follow-up; 29.2% reported at least 1 visit at baseline but no visit at follow-up; 13.5% reported no visit at baseline but at least 1 visit at follow-up; and 24.0% reported at least 1 visit at both times. The follow-up HAMD scores were not normally distributed according to the Shapiro-Wilk test (W = 0.83, p < .001); thus, comparisons between ED visits and HAMD scores were assessed using robust regression. The follow-up HAMD scores for those who had had ED visits at both baseline and follow-up were marginally higher than those that had not used ED (χ^2^ = 3.73, p = .053), but the HAMD scores were significantly higher than those who had reported ED visits only at baseline (χ^2^ = 8.89, p = .003) and those who had used ED only during the follow-up (χ^2^ = 8.64, p = .003). Reduction in HAMD scores since baseline did not differ between ED users at both baseline and follow-up and those who had not used ED (χ^2^ = 0.01, p = .938) or those who had had ED visits only during the follow-up (χ^2^ = 3.62, p = .057).

**Table 3:** ED visit pattern and HAMD score change (n=96)

**ED visit pattern**	**n (%)**	**Baseline HAMD Scores**	**24-week-follow-up HAMD score**	**HAMD score change from baseline**
No visit at both times	32 (33.3)	22.63+5.97	15.03+7.33	-7.59+8.55
Visit at baseline only	28 (29.2)	24.89+7.0	13.0+8.4	-11.89+7.43
Visit at follow-up only	13 (13.5)	23.23+7.76	11.08+5.15	-12.15+8.68
Visit at both times	23 (24.0)	27.30+6.29	19.70+8.14	-7.61+7.09

Table 4 lists the participants’ self-reported reasons for ED visits. At both baseline and follow-up, body pain (not from fall injury and not including chest pain) was the most common reason, followed by breathing problems, chest pain, fall-related injuries, high blood pressure, abdominal pain/intestinal trouble, and infection. Further analysis showed that a significant proportion of high-frequency (3+ visits) users visited the ED for the same reason over time. Specifically, 52% of the high-frequency users at baseline and 56% of those at 24-week follow-up reported the same reasons for at least 3 or more visits: body pain, stomach pain, chest pain, congestive heart failure, falls, breathing problems, high blood pressure, hyperglycemia, seizures, urinary tract infection, wound care, and cancer-related problems. Some participants reported that their calls to their PCPs or on-call nurses during evening hours and weekends (when their PCPs were not present in the clinics) were almost always responded with an instruction to go to an ED. An absolute majority of those who used ED services reported that they would have preferred seeing their PCPs to an ED visit given the long wait in the crowded waiting area in most occasions.

**Table 4:** **Self-reported reasons for ED visits (n,** % **of all visits)**

**Reason**	**Baseline**	**24-week follow-up**
Total number of visits	170 (100)	88 (100)
Body pain not from fall injury and not chest pain	31 (18.2)	11(12.5)
Breathing problem	19 (11.2)	2 (2.3)
Chest pain	19 (11.2)	5 (5.7)
CHF	3 (1.8)	1 (1.1)
Injury from fall	16 (9.4)	7 (8.0)
High blood pressure	13 (7.6)	7 (8.0)
Hypotension	1 (0.5)	3 (3.4)
Abdominal pain/intestinal trouble	11 (6.4)	6 (6.8)
Infection		
Pneumonia/bronchitis	3 (1.8)	6 (6.8)
Urinary tract infection	2 (1.2)	7 (8.0)
Kidney infection	3 (1.8)	2 (2.3)
Pancreas	1 (0.6)	1 (1.1)
Gall bladder		1 (1.1)
Hypo/Hyperglycemia	8 (4.7)	1 (1.1)
Got sick—Not feeling well	7 (4.1)	4 (4.5)
Passed out/seizure	6 (3.5)	1 (1.1)
Cancer-related problems (burning, breathing, and vomiting)	6 (3.5)	
Stroke	3 (1.8)	3 (3.4)
Migraine	3 (1.8)	1 (1.1)
Dehydration	3 (1.8)	
Leg swelling/would not move	3 (1.8)	3 (3.4)
Dizziness/Anemia	2 (1.2)	
Wound care		11 (12.5)
Low blood count	2 (1.2)	
Cold/coughing/throat problem	1 (0.6)	2 (2.3)
Drug interaction/amnesia	1 (0.6)	1 (1.1)
Stress reaction/anxiety	1 (0.6)	1 (1.1)
Car accident		1 (1.1)
Popped vein in scrotum	1 (0.6)	
Itching back	1 (0.6)	

### Association between ED visits and depressive symptoms: multivariable analysis

Preliminary analyses of covariates in the logistic portion of the zero-inflated Poisson model for baseline ED visits indicated that Hispanic status, family support, and the number of medical conditions met the inclusion criteria based on the decision rules described above. The same analyses for the zero-inflated Poisson model for ED visits at 24-week follow-up indicated that Hispanic status, being in the 60–69 age group, and the number of medical conditions met the inclusion criteria based on the decision rules described above. Table 5 shows that the ED visit frequency reported at baseline was significantly positively associated with the baseline HAMD score, 50–59 years of age, 60–69 years of age, and being Hispanic. In addition, family support was a significant predictor in the logistic portion of the model (χ^2^ = 5.97, p = .015). Gender, being Black, Medicaid coverage, number of chronic medical conditions, and pain frequency were not significant correlates of the baseline ED visit frequency.

**Table 5:** Association between depressive symptom severity and ED visit frequency: multivariable analysis results

**Variable**	**Baseline (n=121)**	**24-Week (n=96)**	**24-Week (n=96)**
	**B (SE)**	**B (SE)**	**B (SE)**
Intercept	-0.63 (0.60)	-1.16 (0.82)*	-1.79 (0.87)*
Gender			
Female	0.12 (0.23)	1.73 (0.48)***	1.74 (0.48)***
(Male)			
Age			
50-59	0.83 (0.25)*	1.40 (0.51)**	1.37 (0.51)**
60-69	0.56 (0.25)*	0.76 (0.44)†	0.74 (0.44)†
(70+)			
Race/ethnicity			
Black	0.31 (0.25)	0.81 (0.52)	0.81 (0.51)
Hispanic	0.58 (0.25)*	0.23 (0.45)	0.22 (0.45)
(Non-Hispanic White)			
Medicaid coverage			
Yes	0.18 (0.19)	0.29 (0.29)	0.27 (0.29)
(No)			
Family support	-0.03 (0.02)†	-0.03 (0.02)	-0.03 (0.02)
No. of medical conditions	-0.04 (0.06)	-0.11 (0.09)	-0.11 (0.09)
Pain frequency	-0.04 (0.03)	-0.14 (0.05)**	-0.14 (0.05)**
Baseline HAMD score	0.05 (0.01)***		0.08 (0.03)**
24-week follow-up HAMD		0.07 (0.02)**	
HAMD score changes—			
baseline to 24 weeks^1^			0.07(0.02)**
-2 Likelihood ratio χ^2^	23.49	22.89	24.00
*df*	13	13	14
Model p value	0.036	0.043	0.046

^1^Higher scores indicate worse symptoms since baseline.

**p < 0.01; *p < 0.05’; †p = 0.051.

The ED visit frequency reported at follow-up was significantly positively associated with the follow-up HAMD score, female gender, 50–59 years of age, and pain frequency and it was marginally significantly associated with being in the 60–69 age group. The ED visit frequency at follow-up was also significantly associated with the HAMD score change since baseline, with lower level of reduction or higher level of increase in HAMD scores predicting higher ED visit frequency.

## Discussion

Depression is a serious problem among older adults who are homebound due to disability [24-27]. Low-income homebound persons are even more vulnerable to depression due to multiple life stressors associated with financial hardship [29]. In this study, we examined longitudinal relationship between depressive symptoms and the frequency of ED visits among low-income homebound older adults, aged 50 years and older, who participated in an RCT of a short-term depression treatment. Since the ED visit data were collected from the participants at three assessment points, they provided a great opportunity to examine the relationship between ED visits and depressive symptoms over time.

This study found that 67.7% of the subjects (who provided data at both baseline and follow-up) used the ED at least once during the approximately 12-month study period and that 61% of the visitors made at least one return visit during the 12-month period. These are much higher visit and return visit rates than those reported in other studies [2,6,7] of older adults’ ED use. Since the subjects of this study were low-income, depressed homebound older adults, their high rate of ED visitation was expected; however, the extremely high rate was still surprising. The self-reported reasons that a significant proportion of high-frequency repeat users visited the ED were for relief of the same symptoms that were pain related (e.g., body pain, stomach pain, chest pain). Another reason for their visits appeared to be due to poor self-care (e.g., breathing problems, high blood pressure, hyperglycemia), suggesting that a significant proportion of these ED visits may have been preventable with more effective routine outpatient care, treatment adherence, and self-management of the symptoms, especially pain. Many older adults did not like going to an ED because it often involved a long wait in a crowded waiting room. Frequent ED visits for ambulatory sensitive care also have significant negative societal impact as ED services are far more costly than outpatient physician visits to the Medicare and Medicaid programs.

The study findings support H1 and H2 in that the ED visit frequency was significantly associated with higher depressive symptoms at both baseline and at 24-week follow-up. As noted [19,20], depressive symptoms may have mimicked or exacerbated the somatic symptoms associated with other chronic medical conditions and contributed to poor self-management of these medical conditions. These homebound older adults with comorbid multiple medical conditions and depression were also likely to have felt urgency to seek emergency medical care for symptoms that might be managed in a primary care setting. The findings also support H3, which posited the relationship between ED visit frequency at follow-up and HAMD score change since baseline, controlling for baseline depressive symptom severity. In addition to depressive symptom severity, the findings show that the 50–59 age group had significantly more ED visits at both baseline and follow-up than the 70+ age group. The 60–69 group was also a marginally significant factor for higher ED visits. Although all the participants had their PCPs, the lack of Medicare coverage for some in the under-65 group may have limited effective routine outpatient care for their chronic medical conditions. As compared with 100% of the 70+ age group, 73% of the 50–59 age group and 71% of the 60–69 age group had Medicare (p = .006). Further research is needed to identify other factors (e.g., treatment adherence and self-management of symptoms) contributing to higher ED visits among younger age groups.

Contrary to the findings of some previous studies, this study did not find the number of medical conditions (and level of disability) to be a significant correlate of the ED visit. The discrepancy between the previous and the present study findings is likely due to the greater homogeneity of our subjects compared with those in the previous study samples with respect to their physical and functional health and their economic status. Although pain was the most frequently cited reason for making an ED visit, pain frequency was associated with making an ED visit only at follow-up, controlling for predisposing, enabling, and other need factors. Hispanics were more likely than participants of other ethnicities to use the ED at baseline but not at follow-up, which may indicate that their level of self-care knowledge and skills was lower than that of the other participants at baseline. For example, one study [46] of low-income older Hispanics with diabetes found that those who received diabetes education were less likely to use the ED than those who had not received the education.

The study had a few limitations. First, the self-reported ED visit data were not validated against the subjects’ medical records. Even with possible underreports due to a recall or response bias in self-reports, however, the ED visit rate was much higher than those reported in other studies. Likewise, the reasons for ED use were not validated against the discharge diagnoses. Although self-reported reasons are important in exploring the users’ perception of their need for an ED visit, discharge diagnoses would have helped us understand the true nature of their conditions that brought them to the ED. Second, we chose the 24-item HAMD cutoff score of 15 that was not validated. As described previously, in choosing the cutoff score, we used an empirical approach based on research data from (1) a study of home healthcare patients with major depression that found the mean baseline 24-item HAMD score to be about 15 [36]; and (2) the PROSPECT study in which the lower bound of the 90% confidence interval of the 24-item HAMD for patients with major depression was also about 15 [37,38]. Finally, the sample size was too small to generalize the findings to other groups of homebound adults.

## Conclusions

Despite these limitations, the study has the following clinical implications: First, repeat ED users who tend to visit the ED for the same reason over time need to be provided with better education on self-management of their chronic conditions, taking into consideration their level of health literacy. Second, given the significant relationship between higher depressive symptoms and ED visit frequency, PCPs and EDs may use ED visits as a cue for depression screening. Third, instituting depression treatment that includes symptom management and problem-solving skills may be important to reduce ED visits among medically ill, homebound adults. Fourth, effective pain management is likely to reduce the need for ED visits among low-income homebound older adults. Clinicians need to educate these older adults about effective pain management strategies and the possible relationship between pain and depressive symptoms. Fifth, further research is needed to identify factors contributing to higher ED use by those who are under 70 years.

## Abbreviations

ED: Emergency department; RCT: Randomized controlled trial; HAMD: Hamilton Rating Scale for Depression; PCP: Primary care physician.

## Competing interests

The authors declare that they have no competing interests.

## Authors’ contributions

All authors contributed to the drafting of this version of the manuscript and agree to submission. NGC, MLB, and MEK: conception and design of the study. NGC and CNM: statistical analysis. NGC, CNM; MLB, and MEK: interpretation. All authors read and approved the final manuscript.

## Pre-publication history

The pre-publication history for this paper can be accessed here:

http://www.biomedcentral.com/1471-244X/12/233/prepub

## References

[B1] Aminzadeh F Dalziel WB Older adults in the emergency department: a systematic review of patterns of use, adverse outcomes, and effectiveness of interventions Ann Emerg Med 2002 39 238 247 10.1067/mem.2002.121523 11867975

[B2] Garcia TC Bernstein AB Bush MA Emergency department visitors and visits: who used the emergency room in 2007? NCHS Data Brief 2010 38 1 8 Hyattsville, MD: National Center for Health Statistics 20487622

[B3] Gruneir A Silver MJ Rochon PA Emergency department use by older adults: a literature review on trends, appropriateness, and consequences of unmet health care needs Med Care Res Rev 2011 68 131 155 10.1177/1077558710379422 20829235

[B4] McCusker J Karp I Cardin S Durand P Morin J Determinants of emergency department visits by older adults: a systematic review Acad Emerg Med 2003 10 1362 1370 10.1111/j.1553-2712.2003.tb00011.x 14644789

[B5] Platts-Mills TF Leacock B Cabanas JG Shofer FS McLean SA Emergency medical service use by the elderly: analysis of a statewide database Prehosp Emerg Care 2010 14 329 333 10.3109/10903127.2010.481759 20507220

[B6] Samaras N Chevalley T Samaras D Gold G Older patients in the emergency department: a review Ann Emerg Med 2010 56 261 269 10.1016/j.annemergmed.2010.04.015 20619500

[B7] McCusker J Cardin S Bellavance F Belzile E Return to the emergency department among elders: patterns and predictors Acad Emerg Med 2000 7 249 259 10.1111/j.1553-2712.2000.tb01070.x 10730832

[B8] Naughton C Drennan J Treacy P Fealy G Kilkenny M Johnson F Butler M The role of health and non-health related factors in repeat emergency department visits in an elderly urban population Emerg Med J 2010 27 683 687 10.1136/emj.2009.077917 20581390

[B9] Himelhoch S Weller WE Wu AW Anderson GF Cooper LA Chronic medical illness, depression, and use of acute medical services among medical beneficiaries Med Care 2004 42 512 521 10.1097/01.mlr.0000127998.89246.ef 15167319

[B10] Goldberg SE Whittamore KH Harwood RH Bradshaw LE Gladman JRF Jones RG The prevalence of mental health problems among older adults admitted as an emergency to a general hospital Age Ageing 2012 41 81 86 10.1093/ageing/afr106 PMC3234074 21890483

[B11] Meldon SW Emerman CL Schubert DSP Moffa DA Gaffney Etheart R Depression in geriatric ED patients: prevalence and recognition Ann Emerg Med 1997 30 141 145 10.1016/S0196-0644(97)70132-X 9250635

[B12] Shah MN Jones CMC Richardson TM Conwell Y Katz P Schneider SM Prevalence of depression and cognitive impairment in older adult emergency medical services patients Prehosp Emerg Care 2011 15 4 11 10.3109/10903127.2010.514093 20977363 PMC2991565

[B13] Hastings SN Whitson HE Purser JL Sloane RJ Johnson KS Emergency department discharge diagnosis and adverse health outcomes in older adults J Am Geriatr Soc 2009 57 1856 1861 10.1111/j.1532-5415.2009.02434.x 19694872 PMC2866093

[B14] Oster A Bindman AB Emergency department visits for ambulatory care sensitive conditions Med Care 2003 41 198 207 12555048 10.1097/01.MLR.0000045021.70297.9F

[B15] Katon WJ Lin E Russo J Unützer J Increased medical care costs of a population-based sample of depressed elderly patients Arch Gen Psychiatry 2003 60 897 903 10.1001/archpsyc.60.9.897 12963671

[B16] Unützer J Schoenbaum M Katon WJ Fan M-Y Pincus HA Hogan D Taylor J Healthcare costs associated with depression in medically ill fee-for-service Medicare participants J Am Geriatr Soc 2009 57 506 510 10.1111/j.1532-5415.2008.02134.x 19175438

[B17] Luppa M Heinrich S Matschinger H Sandholzer H Angermeyer MC König HH Riedel-Heller SG Direct costs associated with depression in old age in Germany J Affect Disorder 2008 105 195 204 10.1016/j.jad.2007.05.008 17568683

[B18] Lee BW Conwell Y Shah MN Baker WH Delavan RA Friedman B Major depression and emergency medical services utilization in community-dwelling elderly persons with disabilities Int J Geriatr Psychiatry 2008 23 1276 1282 10.1002/gps.2063 18613268 PMC2587519

[B19] Katon W Ciechanowski P Impact of major depression on chronic medical illness J Psychosom Res 2002 53 859 863 10.1016/S0022-3999(02)00313-6 12377294

[B20] Padget DK Brodsky B Psychological factors influencing non-urgent use of the emergency room: a review of the literature and recommendations for research and improved service delivery Soc Sci Med 1992 35 1189 1197 10.1016/0277-9536(92)90231-E 1439937

[B21] United States Census Bureau: Table 35 Persons 65 Years and Over—Living Arrangements and Disability Status Statistical Table Based on data from the 2009 American Community Survey 2009 http://www.census.gov/compendia/statab/2012/tables/12s0035.pdf

[B22] Anderson G Chronic conditions: Making the case for ongoing care 2010 Robert Wood Johnson Foundation 1 43 http://www.rwjf.org/content/dam/web-assets/2010/01/chronic-care

[B23] Qiu WQ Dean M Liu T George L Gann M Cohen J Bruce ML Physical and mental health of homebound older adults: an Overlooked population J Am Geriatr Soc 2010 58 2423 2428 10.1111/j.1532-5415.2010.03161.x 21070195 PMC3044592

[B24] Bruce ML McVay J Raue PJ Brown EL Meyers BS Keohane DJ Jagoda DR Weber C Major depression in elderly home health care patients Am J Psychiatry 2002 159 1367 1374 10.1176/appi.ajp.159.8.1367 12153830

[B25] Cohen-Mansfield J Shmotkin D Hazan H The effect of homebound status on older adults J Am Geriatr Soc 2010 58 2358 2362 10.1111/j.1532-5415.2010.03172.x 21087220 PMC3119862

[B26] Ell K Unützer J Aranda M Sanchez K Lee P-J Routine PHQ-9 depression screening in home health care: depression prevalence, clinical and treatment characteristics, and screening implementation Home Health Care Services Quart 2005 24 1 19 10.1300/J027v24n04_01 PMC1421520 16446263

[B27] Sirey JA Bruce ML Carpenter M Booker D Reid MC Newell KA Alexopoulos GS Depressive symptoms and suicidal ideation among older adults receiving home-delivered meals Int J Geriatr Psychiatry 2008 23 1306 1311 10.1002/gps.2070 18615448 PMC3634570

[B28] Centers for Medicare and Medicaid Services Medicare and Medicaid Supplemental Statistics Table 7.1 Trends in Persons Served, Visits, Total Charges, Visit Charges, and Program Payments for Medicare Home Health Agency Services: Selected Calendar Years 1974–2010 2011 http://www.cms.gov/Research-Statistics-Data-and-Systems/Statistics-Trends-and-Reports/MedicareMedicaidStatSupp/2011.html

[B29] Choi NG Hegel MT Marinucci ML Sirrianni L Bruce ML Association between participant-identified problems and depression severity in problem-solving therapy for low-income homebound older adults Int J Geriatr Psychiatry 2012 27 491 499 10.1002/gps.2741 21638330 PMC3196815

[B30] Proctor EK Hasche L Morrow-Howell N Shumway M Snell G Perceptions about competing psychosocial problems and treatment priorities among older adults with depression Psychiat Serv 2008 59 670 675 10.1176/appi.ps.59.6.670 PMC3412406 18511588

[B31] Andersen RM Revisiting the behavioral model and access to medical care: does it matter? J Health Soc Behav 1995 36 1 10 10.2307/2137284 7738325

[B32] Centers for Medicare and Medicaid Services Definition of Homebound http://www.medicare.gov/homehealthcompare/Resources/Glossary.aspx?toolAudiance=HHC&Language=English&TermID=0026

[B33] Kroenke K Spitzer RL The PHQ-9: a new depression diagnostic and severity measure Psychiat Ann 2002 32 509 515

[B34] Kroenke K Spitzer RL Williams JB The PHQ-9: validity of a brief depression severity measure J Gen Inter Med 2001 16 606 813 10.1046/j.1525-1497.2001.016009606.x PMC1495268 11556941

[B35] Choi NG Teeters M Perez L Farar B Thompson D Severity and correlates of depressive symptoms among recipients of meals in wheels: age, gender, and racial/ethnic difference Aging Ment Health 2010 14 145 154 10.1080/13607860903421078 19946802

[B36] Bruce M Brown E Raue P Mlodzianowski A Meyers B Leon A Heo M Byers A Greenberg R Rinder S Katt W Nassisi P A randomized trial of depression assessment intervention in home health care J Am Geriatr Soc 2007 55 1973 1800 10.1111/j.1532-5415.2007.01419.x 17916119

[B37] Bruce ML Ten Have T Reynolds CF Katz I Schulberg HC Mulsant BH Brown GK McAvay GJ Alexopoulos GS Reducing suicidal ideation and depressive symptoms in depressed older primary care patients: a randomized controlled trial JAMA 2004 291 1081 1091 10.1001/jama.291.9.1081 14996777

[B38] Alexopoulos GS Reynolds CF 3rd Bruce ML Katz IR Raue PJ Mulsant BH Oslin DW Ten Have T PROSPECT Group Reducing suicidal ideation and depression in older primary care patients: 24-month outcomes of the PROSPECT study Am J Psychiatry 2009 166 882 890 10.1176/appi.ajp.2009.08121779 19528195 PMC2838419

[B39] Borson S Scanlan J Brush M Vitaliano P Dokmak A The Mini-Cog: a cognitive “vital signs” measure for dementia screening in multi-lingual elderly Int J Geriatr Psychiatry 2000 25 1021 1027 11113982 10.1002/1099-1166(200011)15:11<1021::aid-gps234>3.0.co;2-6

[B40] Wallihan DB Stump TE Callahan CM Accuracy of self-reported health services use and patterns of care among urban older adults Med Care 1999 37 662 670 10.1097/00005650-199907000-00006 10424637

[B41] Lubben JE Gironda MW Osterweil D, Brummel-Smith K, Beck JC Social support networks Comprehensive Geriatric Assessment 2000 New York: McGraw-Hill 127 137

[B42] Depression Rating Scale Standardization Team GRID-HAMD-17, GRID-HAMD-21 Structured Interview Guide 2003 San Diego, CA: International Society for CNS Drug Development

[B43] Moberg PJ Lazarus LW Mesholam RI Bilker W Chuy IL Neyman I Markvart V Comparison of the standard and structured interview guide for the Hamilton Depression Rating Scale in depressed geriatric inpatients Am J Geriatr Psychiatry 2001 9 35 40 11156750

[B44] Hosmer DW Lemeshow S Applied Logistic Regression (2nd editon) 2000 New York: John Wiley and Sons

[B45] Burnham KP Anderson DR Model selection and multimodel inference 2002 2 New York: Springer

[B46] Mier N Wng X Smith ML Irizarry D Treviño L Alen M Ory MG Factors influencing health care utilization in older Hispanics with diabetes along the Texas-Mexico border Popul Health Manag 2012 15 149 156 10.1089/pop.2011.0044 22313441 PMC3429293

